# Anti-Depressant Properties of Crocin Molecules in Saffron

**DOI:** 10.3390/molecules27072076

**Published:** 2022-03-23

**Authors:** Shahida Anusha Siddiqui, Ali Ali Redha, Edgar Remmet Snoeck, Shubhra Singh, Jesus Simal-Gandara, Salam A. Ibrahim, Seid Mahdi Jafari

**Affiliations:** 1 Campus Straubing for Biotechnology and Sustainability, Technical University of Munich, Essigberg 3, 94315 Straubing, Germany; s.siddiqui@dil-ev.de; 2 German Institute of Food Technologies (DIL e.V.), 49610 D-Quakenbrück, Germany; 3 Department of Sport and Health Sciences, College of Life and Environmental Sciences, University of Exeter, Exeter EX1 2LU, UK; ali96chem@gmail.com; 4 Centre for Nutrition and Food Sciences, Queensland Alliance for Agriculture and Food Innovation (QAAFI), The University of Queensland, Brisbane, QLD 4072, Australia; 5 Food Technology Study Programme, HAS University of Applied Sciences, Onderwijsboulevard 221, 5223 DE ‘s-Hertogenbosch, The Netherlands; edgar.snoeck@gmail.com; 6 Department of Tropical Agriculture and International Cooperation, National Pingtung University of Science and Technology, No. 1, Xuefu Rd, Neipu Township, Pingtung City 912, Taiwan; shubhrasingh6@gmail.com; 7 Nutrition and Bromatology Group, Department of Analytical Chemistry and Food Science, Faculty of Science, Universidade de Vigo, E-32004 Ourense, Spain; jsimal@uvigo.es; 8 Food and Nutritional Sciences Program, North Carolina Agricultural and Technical State University, E. Market Street, 1601, Greensboro, NC 24711, USA; ibrah001@ncat.edu; 9 Faculty of Food Science and Technology, Gorgan University of Agricultural Sciences and Natural Resources, Gorgan 49189, Iran

**Keywords:** *Crocus sativus*, saffron, crocin, natural anti-depressants, traditional medicine

## Abstract

Saffron is a valued herb, obtained from the stigmas of the *C. sativus* Linn (Iridaceae), with therapeutic effects. It has been described in pharmacopoeias to be variously acting, including as an anti-depressant, anti-carcinogen, and stimulant agent. The therapeutic effects of saffron are harbored in its bioactive molecules, notably crocins, the subject of this paper. Crocins have been demonstrated to act as a monoamine oxidase type A and B inhibitor. Furthermore, saffron petal extracts have experimentally been shown to impact contractile response in electrical field stimulation. Other research suggests that saffron also inhibits the reuptake of monoamines, exhibits *N*-methyl-d-aspartate antagonism, and improves brain-derived neurotrophic factor signaling. A host of experimental studies found saffron/crocin to be similarly effective as fluoxetine and imipramine in the treatment of depression disorders. Saffron and crocins propose a natural solution to combat depressive disorders. However, some hurdles, such as stability and delivery, need to be overcome.

## 1. Introduction

Dried stigmas of the perennial flower *Crocus sativus* Linn (Iridaceae) produce a valued herb: saffron. Dubbed as “red gold” and “golden condiment”, saffron has been named in cookbooks and pharmacopoeias throughout history and geography, including Ebers papyrus (Egyptian, 1550 BC), Apicius (Roman, 1st century), Materia Medica (Greek, 1st century), Avicenna’s Canon of Medicine (Persian, 11th century), and Indian Ayurvedic literature [1,2,3,4,5]. Furthermore, it also serves as a dye in food products and textiles and as an aromatic in perfumes and cosmetics. These dried floral constituents most often are the vibrant stigmas of the flower, but sometimes also include styles and other floral tissue (e.g., filaments) [6]. The herb is of a red color and has a bitter taste and a pleasant fragrance. It has been employed in traditional medicine as an anti-depressant, anti-carcinogen, and stimulant, along with a great host of other functions [1,7].

Saffron is presently cultivated in Iran, India, Spain, Russia, and many other countries [6,7]. Geography varies widely, spanning Eurasia and including some of Africa. The saffron is sterile through its polyploidy, and propagation only occurs vegetatively via the daughter corms. Because of this asexual reproduction, no breeding efforts can be employed, limiting improvements to the selection of advantageously mutated corms. Only slight morphological and biochemical differences exist between these clones geographically. Morphological abnormalities rarely occur. This usually manifests in more or less than three-branched stigmas, through the fusion of flowering buds [8]. Saffron’s value is tied to its limited production methods. Harvesting is a laborious manual process and difficult to mechanize [9,10]. Saffron flowers in the autumn. Harvesting is only possible upon first bloom, as the frost of the superseding night will damage the flower. Therefore, harvesting is only done on a per flower basis. The subsequent separation of the styles and stigmas is most often also manual work, where the worker must regard not damaging the herb.

Ancient civilizations recognized saffron’s multitude of therapeutic effects. Second millennium B.C. Assyrians and Babylonians employed saffron as a medicine against dyspnea, neurological disorders, menstruation, and painful urination [11]. The Greeks used saffron against insomnia, addiction withdrawal, and hangovers [1]. The Egyptians used it as an incense with sedative qualities [12]. Saffron’s therapeutic effects are copious, as will become apparent from Table 1.

The present study provides an overview of saffron and its constituent crocin as an anti-depressant in historic medicine and the respective modern evidence. To this end, Scopus and Google Scholar were queried for reports on the pharmacological activities of saffron constituents, with particular focus on crocin, relating to mechanisms pertaining to depressive disorders. Additionally, the bioavailability of crocins and delivery mechanisms were investigated.

## 2. Depression and Associated Disorders and Role of Natural Products as Adjunct Therapy

Depressive disorders are a group of emotional states centering around sadness. These range in severity, varying between unhappiness and discontent to a debilitating state of despondency [28]. Two predominant depression disorders are persistent depressive disorder (PDD) and major depressive disorder (MDD). PDD, or dysthymia, typically endures longer but with less severity than MDD. A variation on MDD, bipolar disorder (or, formerly, manic depression) is accompanied by episodic mania [29]. Other types of depression are recognized, e.g., perinatal depression, seasonal affective disorder, and psychotic depression. Depression severities are commonly classed with the aid of the Hamilton Depression Rating Scale (HAM-D) [30].

The mechanisms pertaining to depression are still theorized but generally point to a deficiency. Depressions are explained through the monoamine theory by a lack of three neurotransmitter molecules: serotonin, norephedrine, and dopamine. Alternatively, the more recent neurogenic theory ascribes a deficiency of neurons to cause depression. Medication, psychotherapy, brain stimulation therapy, or a combination thereof can be employed as curatives [31]. Pharmacological treatments aim to either inhibit neurotransmitter reabsorption (e.g., selective serotonin reuptake inhibitors (SSRIs), selective serotonin and noradrenaline reuptake inhibitors (SSNRIs), or tricyclic anti-depressants (TCAs)) or inhibit neurotransmitter-degrading enzymes (monoamine oxidase (MAO)), distinguishable in MAO isoform A (affinitive to serotonin and somewhat norepinephrine) and B (acting strongly on phenylethylamine and benzylamine) [32,33].

Depressive disorders have consistently had a large impact on global DALYs. This burden has increased most recently, exacerbated by the COVID-19 pandemic [34]. Natural officinal remedies have been reported throughout history. For instance, traditional Chinese medicine has employed *Panax ginseng* root for millennia as a mood enhancer [35,36]. Xu et al. [37] found that ginsenosides, particularly (S)-protopanaxadiol, exhibited strong anti-depressant effects in rats. Similarly, peony extract, a derivative of the *Paeonia lactiflora* root, was utilized as an anti-depressant in traditional Chinese medicine. Indeed, peony has been shown to exhibit anti-depressant-like effects in stressed rodents [38]. Moreover, the seeds of the *Ginkgo biloba* have been employed in traditional Chinese medicine for their neuro-protective effects [39]. Curcuma has been incorporated in both traditional Indian and Chinese medicine in an effort to regulate stress and mood disorders [40,41]. Chlorophytum comosum has traditionally been used in traditional medicinal preparations in India, China, and Africa, with the constituent stigmasterol exerting neuroprotective effects [42].

Tea spread from China to Japan as medicine, being later described in the Japanese book Kissa Youjouki as a marvelous medicine preventive for many ailments [43]. Catechins, present in green tea, have experimentally been shown to act as a possible MAO inhibitor in mice [44,45]. Similarly, many other plant molecules/extracts have been found to be psychotropic in experimental models (Table 2).

## 3. Saffron: Reported Biologically Active Compounds and Their Pharmacology

Saffron herb is host to a plethora of bioactive compounds including carotenoids (crocetin, crocins, α-carotene, lycopene, and zeaxanthin), monoterpene aldehydes (e.g., picrocrocin and safranal), monoterpenoids (e.g., crocusatines), isophorones, and flavonoids [2,55]. Crocetin and its glycosidic analogues crocin, picrocrocin, and safranal are regarded as the most notable bioactive molecules [56]. A myriad of pharmaco-active functions is attributed to these compounds.

**Figure 1:**
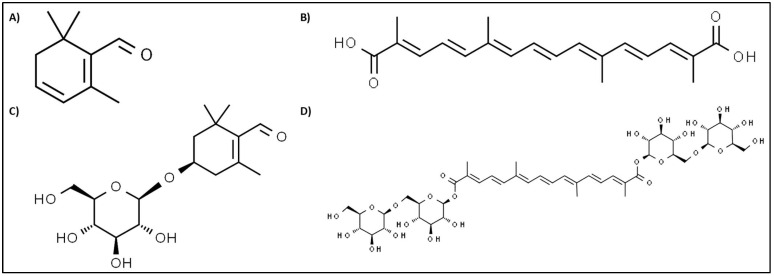
Structural formulas of saffron constituents safranal (**A**) [61], trans-crocetin (**B**) [62], picrocrocin (**C**) [63], and trans-crocetin digentiobiose ester (**D**) [64], one of crocin’s many forms [65].

Saffron’s aroma is chiefly attributed to the volatile compound safranal (Figure 1A). Safranal attenuated oxidative damage induced through cerebral ischemia in rats [57]. Research has found safranal to act on neurological disorders. For instance, safranal proved to be an effective anti-convulsant in mice, whereas crocin did not [58]. Similarly, Hosseinzadeh and Sadeghnia [59] found safranal to be protective against seizures in rats. Other studies on mice have attributed anti-depressant properties to safranal and crocin via the mechanism of inhibiting dopamine, serotonin, and norepinephrine reuptake [60,61].

Crocetin (Figure 1B) and crocins were shown to inhibit in vivo and in vitro angiogenesis, with crocetin being more effective [66]. Thus, crocetin could possibly be employed to retard abnormal blood vessel growth. Furthermore, crocetin has been shown to be anti-carcinogenic. Its mechanisms include the inhibiting synthesis of nucleic acid, enhancement of anti-oxidative systems, apoptosis initiation, and growth hindrance of signaling pathway factors [67]. Conflictingly, Escribano et al. [68] attributed no cytotoxic effect to crocetin, whereas the other three compounds did inhibit cell growth.

Crocins, the molecules of subject in this paper, are carotenoids jointly responsible for saffron’s vibrant color. Several of saffron’s curative functions can be related to this group of compounds. It has acute and chronic anti-inflammatory effects. This has been demonstrated in both in vitro cyclooxygenase inhibition assays and in vivo tests with edemas in rodents [69]. Moreover, it has in vivo been shown to relieve cerulein-induced pancreatic inflammation [70]. Furthermore, crocins can alleviate neurological disorders. Georgiadou et al. [71] alleviated manually induced schizophrenia-like behavior in rats by administering crocins. Lastly, crocins exhibited anti-depressant activity through neurotransmitter reuptake inhibition. This has been demonstrated in vivo and in vitro [61,72,73]. Notably, crocetins are more readily absorbed than crocins in the gastrointestinal tract of animals [74]. Additionally, crocins are metabolized to crocetins when administered orally [74,75,76]. However, it has not yet been elucidated how readily crocin is metabolized in humans. Nevertheless, the method of administration must be significant for pharmacokinetics.

Picrocrocin (Figure 1C), a colorless, bitter-tasting compound, shares therapeutic effects with the other three compounds (e.g., anti-carcinogenic) [68]. However, to the best of our knowledge, isolated picrocrocin studies are limited and its role as a neuroprotective agent has not been described yet [77].

## 4. Role of Saffron Stigma Extract and Crocin in Synaptic Transmission

Crocins are natural carotenoids, commercially obtained from the dried stigma of saffron, occurring with different esterified saccharides on a crocetin backbone, such as *trans*-crocetin (β-d-glucosyl)-(β-d-gentiobiosyl) ester (named *trans*-3-Gg), *trans*-crocetin di-(β-d-glucosyl) ester (named *trans*-2-gg), *trans*-crocetin di-(β-d-gentiobiosyl) ester (named *trans*-4-GG; Figure 1D), *trans*-crocetin (β-d-gentiobiosyl) ester (named *trans*-2-G), *cis*-crocetin (β-d-glucosyl)-(β-d-gentiobiosyl) ester (named *cis*-3-Gg), and *cis*-crocetin di-(β-d-gentiobiosyl) ester (named *cis*-4-GG). Saffron’s brick-red color is generally a result of the glycoside carotenoid structure of crocin [78]. Moreover, the main interest in this herb could be due to its anti-anxiety, anti-convulsant, and hypnotic properties. It is believed that bioactive compounds such as crocin, crocetin, and others are attributed for their anti-oxidant properties, which may partly justify their neuroprotective effects [79].

Several studies have demonstrated that saffron not only inhibits the reuptake of monoamines but also exhibits both *N*-methyl-d-aspartate (NMDA) receptor antagonism and γ-aminobutyric acid agonism, which seem to be responsible for its anti-depressant-like and anxiolytic effects demonstrated in animal models [80]. It was concluded from the human and animal studies that saffron, mainly crocin, has shown a positive effect in the treatment of mild to moderate depression, which might be possibly due to the interaction of serotonin and the noradrenaline system [81].

According to the studies of various parts of the saffron flower, contractile responses to electrical field stimulation (EFS) in isolated vas deferens in rats were reduced by saffron petal extracts. The contractions of EFS-induced vas deferens were shown to be mediated by noradrenaline and adenosine triphosphate from sympathetic nerves. The ethanolic extract of saffron was noted to show changes in EFS in rats’ isolated vas deferens; however, the aqueous extract of the saffron was more effective in guinea pig ileum [82]. Saffron and crocin were found to have an inhibitory impact on amyloid beta-peptide fibrillogenesis and a protective action against H_2_O_2_-induced toxicity in human neuroblastoma cells in an in vitro study. Saffron (60 mg/kg body weight, i.p.) significantly increased learning and memory in normal and old mice after a week of administration, demonstrating cognitive-enhancing properties [83]. In another study, crocin activity was linked with reactive oxygen species’ production and causing oxidative stress, for instance, by the treatment with 5 and 25 mg/mL of saffron extract; 10 and 50 μM of crocin lowered the neurotoxic effect of glucose in ROS-mediated PC12 cells [84].

Some clinical studies have shown that in a randomized and double-blind study, saffron supplementation statistically improved the mood of subjects compared to the placebo group. For 6 weeks, the administration of saffron extract (30 mg/day) was effective in the treatment of mild to moderate depression based on the HAM-D. These effects were similar to the effects of fluoxetine, which is an anti-depressant known as an SSRI [85,86]. The therapeutic benefits of petals of saffron in the treatment of mild to moderate depression have also been suggested [87]. The efficacy of the co-administration of a hydro-alcoholic extract of saffron (40 or 80 mg) and fluoxetine (30 mg/day) was also investigated in a double-blind, randomized clinical trial for 6 weeks. The results revealed that a dose of saffron of 80 mg plus fluoxetine was more effective to treat mild to moderate depressive disorders than that of saffron of 40 mg and fluoxetine [88].

## 5. Monoamine-Related Mechanism and Brain Neurotransmitters

All the anti-depressant drugs increase the monoamine concentration in the brain; therefore, it is important to note that depression is defined by the serious condition of brain monoamine reduction [89]. The MAO inhibitory properties of crocin and safranal were evaluated to assess their influence on catecholamine and 5-HT levels in the brain. In particular, crocin was demonstrated to be a non-competitive inhibitor of the human MAO-A and MAO-B in the micromolar range by means of binding to allosteric sites on the enzyme, whereas safranal was inactive toward both isoforms. It is known that MAO-A and MAO-B are two important enzymes that are targets for the treatment of neurodegenerative disorders [90]. Saffron extract co-administered with aluminum induced changes in MAO (A and B) activity and the levels of lipid peroxidation in the whole brain and cerebellum [81].

The emergence of depression is associated with several physiological disturbances in monoaminergic activity, hypothalamus-pituitary-adrenal activity, inflammation, and oxidative and nitrosative stress [91]. Crocin is a group of hydrophilic carotenoids consisting of an esterified monoglycosyl or disaccharide gentiobiose on the dicarboxylic acid crocetin [92]. The possible molecular mechanisms suggest that crocin exerted anti-inflammatory activity in a rabbit osteoarthritic model by inhibiting interleukin 1 beta-induced activation of the nuclear factor-kappa B NF-kB pathway [93]. Furthermore, crocin decreased the mRNA expression of tumor necrosis factor α (TNF-α), IL-1β, IL-6, interferon-γ (IFN-γ), NF-κB, cyclooxygenase-2 (COX-2), and inducible nitric oxide synthase (iNOS) [94].

In another hypothesis of the mechanism, brain-derived neurotrophic factor (BDNF) is a member of the neurotrophin superfamily, which includes growth factors that promote learning and memory by cell survival, differentiation, and death of specific neuronal populations. The epigenetic modulation of BDNF and TRKb genes might contribute to the pathophysiology of depression and related behaviors [93,95]. The anti-depressant-like activity increases the cyclic adenosine monophosphate (cAMP) response element-binding protein (CREB), BDNF, and VGF levels in the hippocampus [96]. Another important family involved in the mechanism belonging to serine/threonine protein kinases is mitogen-activated protein kinases (MAPK), which regulate neuronal activity and synaptic plasticity. Activation of the MAPK cascade requires four sequential events, which include small GTPases (Ras and Rac proto-oncogenes), MAPK kinase kinases (Raf or MEKK), MAPK kinases (MEK), and MAPKs. The activation of the MAPK cascade results in linking the extracellular signals to synaptic responses [96]. The Ras-Raf-MEK1/2 pathway is responsible for activating extracellular signal-regulated kinase (ERK), which plays a pivotal role in psychiatric disorders including depression and anxiety [97]. High concentrations of crocin significantly reduced p-MEK. Therefore, modulation in the BDNF/CREB/ERK signaling cascade and inhibition through crocin might provide further insights into the importance of behavioral changes during the depression [96].

**Figure 2:**
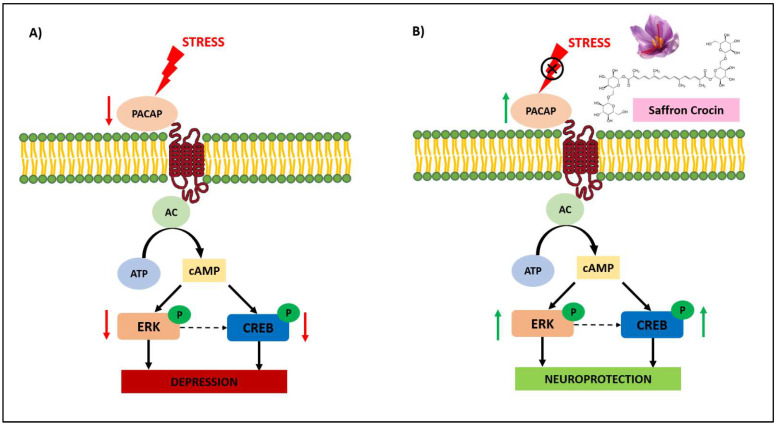
Mechanism for the neuroprotective effect of crocin in depression. Stress can cause depression (**A**), however, saffron crocin can reduce the effect of stress by exhibiting neuroprotection activity (**B**). PACAP, pituitary adenylate cyclase-activating polypeptide; ERK, extracellular regulated protein kinases; CREB, response element binding protein; cAMP, cyclic adenosine monophosphate; ATP, adenosine triphosphate; AC, adenylyl cyclase [98].

The expression of pituitary adenylate cyclase-activating polypeptide (PACAP) is inhibited by stress, which results in the inhibition of the phosphorylation of extracellular regulated protein kinases (ERK) and response element binding protein (CREB), and results in the reduction of the translation of synaptic plasticity proteins, which untimely causes depression, as shown in Figure 2A [98]. Saffron crocin can upregulate endogenous PACAP, resulting in the activation of ERK and CREB. This will improve synaptic plasticity and enhance the neuronal survival, as shown in Figure 2B. This mechanism has been reported based on mice and corticosterone cell models.

The role of the saffron extract is involved in inhibiting serotonin reuptake in synapses, thereby enhancing its positive effects while combating depression. To date, research suggests that the reuptake inhibition of monoamines, MAO inhibition, NMDA antagonism, and improved brain-derived neurotrophic factor signaling may be mechanistic factors responsible for the treatment of depression from saffron [99].

## 6. Neurotransmitter Receptors and Possible Targets for Crocin

**Figure 3:**
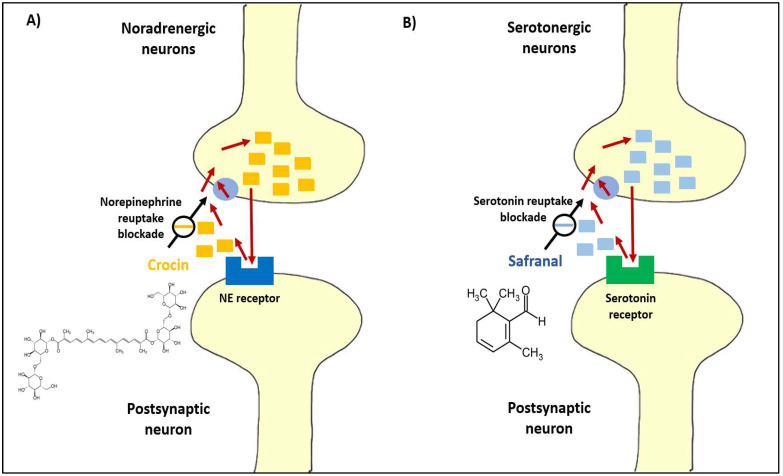
Illustration of the mechanism of inhibiting of norepinephrine (NE) reuptake and serotonin reuptake by (**A**) crocin and (**B**) safranal, respectively [101].

The cholinergic synapses present in the human central nervous system are responsible for the transmission of critically important brain functions such as memory, learning, attention, etc. [81]. Anti-depressants are reported to function by triggering serotonin, norepinephrine, and dopamine levels in the brain. To confirm this, Ettehadi et al. [100] measured changes in rat brain dopamine, serotonin, norepinephrine, and glutamate concentrations; after the administration of an aqueous extract of saffron (50, 100, 150, and 250 mg/kg, i.p.), saffron increased brain dopamine concentration in a dose-dependent manner. In addition, the results showed that the aqueous extract of saffron, especially at the dose of 250 mg/kg, increased the production of important neurotransmitters including dopamine and glutamate in rat brain [100]. In fact, it was reported based on animal studies that the possible anti-depressant activity of saffron bioactive compounds (crocin and safranal) could be mainly through inhibiting serotonin reuptake and the inhibition of dopamine and norepinephrine reuptake (Figure 3) [101].

The effects of saffron on conditioning place preference induced by morphine have been reported to be similar to the effect of NMDA receptor antagonists [102]. Therefore, an interaction of saffron and glutamate receptors in the nervous system might be postulated in glutamatergic system. The high calcium permeability through NMDA receptors plays an important role in post-training memory processing by the amygdala and hippocampus parts of the brain [103]. Similarly, the cholinergic system imparting learning and other higher brain functions in the central nervous system have also been mediated by the effect of saffron extract [104,105]. Therefore, there are requirements for more mechanistic-based experiments to study the involvement of saffron extract in cognition and depression.

### 6.1. Serotonin

It was observed from the abovementioned studies considering a biochemical point of view that crocin from saffron is proven to be able to find a balance with a highly complex mechanism with some of the major neurotransmitters, such as in serotonergic activity, in which each type of neurotransmitter carries specific information and creates a unique set of an individual’s brain chemistry. In addition to that, saffron is reported to modulate the hypothalamus-pituitary-adrenal (HPA) axis and imparts a neuroprotective effect. Saffron is reported to increase the levels of superoxide dismutase, catalase, and glutathione peroxidase, while lowering malondialdehyde levels and inhibiting the lipid peroxidation pathway. Moreover, saffron positively influences brain plasticity in which the ability of neural networks increases through growth and reorganization [106].

### 6.2. Dopamine

To identify cellular and molecular mechanisms underlying the anti-depressant property of saffron, it is important to measure another important neurotransmitter commonly responsible for creating positive feelings associated with reward or reinforcement activity. In recent studies, saffron was responsible for treating mild to clinical depression. The findings suggested that an aqueous extract of saffron contains an active component that possibly accelerates dopamine and glutamate levels in the brain suffering with depression [100]. Due to the complex anatomy of the brain, it is difficult to investigate whether the dopamine and glutamate are released in the vesicles or in the synaptic space; however, both the aqueous and organic extracts of saffron showed significant results in reducing symptoms of depression.

## 7. Pharmacological Treatment of Depression with Crocin

Several pharmacological activities have been suggested to be involved in the anti-depressant-like effects (Table 3). In the following section, we discuss these potential treatments and effects of saffron on mild, moderate, and major depression. The emerging interest in herbal medicine for depression will eventually replace the long-standing reliance on synthetic anti-depressants; for example, saffron has gained a reputation to be used as a natural source to fight the symptoms of depression. The studies showed the effect of saffron’s stigma was as effective as chemically derived anti-depressants such as imipramine and fluoxetine in mild to moderate depression [85,86]. Similarly, saffron was equally effective as citalopram in the major depressive disorder with anxious distress [107] and decreased mild to moderate generalized anxiety disorder when compared with sertraline [108].

Other parts of saffron such as petals proved to be effective on the HAM-D in the treatment of depression [109]. In addition, comparing the results in depressed adult outpatients, it was concluded that the petals of saffron were as effective as the synthetic antidepressant fluoxetine [87]. Even in a randomized, clinical trial, fluoxetine was given with the regulated amount of saffron (40 and 80 mg/day) and showed promising results in the treatment of mild to moderate depression [88]. Saffron significantly decreased the mild to moderate depression in those with post-menopausal hot flashes when compared to fluoxetine [110].

Saffron stigma was shown to reduce mild to moderate post-partum depression in mothers [111]. It was also found to be effective during mild to moderate depression in patients suffering from post-percutaneous coronary intervention [112]. Likewise, an aqueous extract of saffron and its crocin was found to significantly improve mild to moderate depression in patients with coronary artery disease [113]. Additionally, there was significant decrease in the treatment of depression during premenstrual syndrome [114]. Crocin showed lower symptoms of depression in subjects with metabolic syndrome [115]. Saffron comparably improved depression and dysfunction such as reduced homocysteine levels in patients with major depression [116]. Affron^®^, a standardized extract from saffron, showed a significant reduction in mild to moderate youth anxiety and depressive symptoms [117].

As mentioned in the above studies, the stigma of saffron showed a significant decrease in mild to moderate depression [118] and the petals of saffron were used to improve signs of major depression [109]. When compared to fluoxetine, saffron reduced depression and improved the lipid profile [120]. Crocin also showed a significant decrease in major depression [121]. Similarly, crocin had effects on psychological parameters in patients under methadone maintenance treatment to improve depression-like symptoms [122]. Results revealed that there is huge potential for accepting saffron as an herbal drug for the treatment of mild to moderate depression; however, more research is required for it to be accepted against major depression. Table 3 depicts an overview of studies employing saffron and crocin as an anti-depressant.

Crocins have been demonstrated to be potentially applicable as an anti-depressant. However, crocins have been found as poorly bioavailable, with a small percentage permeating the digestive tract [124]. Furthermore, crocins are deglycosylatized into crocetin through hydrolysis when orally ingested [56,124,125]. Intra-peritoneal injection does allow unaltered crocins to penetrate the blood–brain barrier [126]. Nevertheless, drug stability and bioavailability should be increased to not hamper the desirable administration route of oral ingestion. Nanocarriers have been demonstrated to be applicable aids in biological delivery processes [119,123,127]. Various matrices have been shown to increase and retain crocins, increasing delivery and stability (Table 4). The exploitation of nanomaterials poses a promising route. However, the efficacy of gastrointestinal tract and blood–brain barrier permeation and crocin hydrolysis remains unspecified in most cases.

## 8. Conclusions and Future Perspectives

For ages, people have preferred the dried stigma of *Crocus sativus* (saffron) for medicinal and nutritional purposes in different parts of the world, mainly in Central Asia, Iran, China, India, Turkey, Algeria, and Europe. The most active biological compounds include crocetin, crocin, picrocrocin, and safranal, which are responsible for color, taste, and fragrance, respectively. These play a pivotal role in the central nervous system associated with anxiety and depression. These bioactive compounds are also neuroprotective and anxiolytic and can benefit learning and memory impairments. The most popular anti-depressants that are prescribed by physicians are tricyclic anti-depressants (TCAs), selective serotonin reuptake inhibitors (SSRIs), and selective serotonin noradrenaline reuptake inhibitors (SSNRIs). The primary mechanism works by enhancing serotonin, a special neurotransmitter.

The high cost of saffron is due to labor-intensive production, which initially requires handpicked stigmas for saffron. These precious stigmas contain all the bioactive compounds that are responsible for its medicinal properties. This existing evidence suggests that saffron has a potential to be used efficaciously to alleviate the symptoms of depression in different conditions from pre-menstrual to post-partum. On the contrary, this statement has to be supported by more clinical evidence and large-scale trials among these areas of study to address methodological limitations and a lack of global diversity in clinical recommendations. A wider perspective can open avenues for a detailed mechanistic approach, proper dosage, more bioactive composition, and the long-term safety in the herbal industry. However, natural compounds have major challenges such as low bioavailability. Furthermore, natural compounds are generally rapidly metabolized and have the ability to cross the blood–brain barrier. Perhaps these challenges could be overcome with the development of nanocarriers that are site specific and target the diseased subset of neurons instead of affecting healthy neurons. The targeted delivery of natural compounds to the affected part of the brain can result in designing a system that includes reduced side effects and the controlled release of particular drugs as well as a higher bioavailability of a drug at a particular site. Therefore, brain delivery of natural inhibitors through a nanocarrier-based approach is futuristic, will contribute to potential CNS therapies, and will allow a novel strategy for saffron in therapeutic applications.

## Figures and Tables

**Table 1:** Reported functions of saffron and its extracts in experimental trials.

Function	Experimental Findings	Reference
Diuretic agent	Doses of 120 and 240 mg/kg BW have been shown to have diuretic activity in rats, however, at lower activity than hydrochlorothiazide.	[13]
Analgesic agent	Safranal, ethanolic, and aqueous saffron extracts acted as analgesic agents in animal models.	[14]
	Aqueous saffron extracts reduced pain in rats during the chronic phase of formalin test, in a dose-dependant manner	[15]
Anti-nociceptive	Aqueous and ethanolic extracts of stigmas and petals reduced pain signaling from acetic acid-induced writhing.	[16]
Anti-inflammatory	Ethanolic saffron stigma extracts exhibited edema inhibition, with similar coagulation time to aspirin.	[17]
	Stigma extracts showed weak to moderate effect against acute xylene inflammation in mice. However, both stigmas and petal extracts exerted anti-inflammatory effects in edema-induced chronic inflammation in rats.	[16]
Anti-convulsant	Aqueous and ethanolic extracts of stigmas retarded the initiation and duration of tonic convulsions in mice.	[18]
Bronchodilatory	Concentrations varying between 4 and 16 mg/mL of saffranal had a preventive effect on the tracheal responses in guinea pigs	[19]
Secretagogues/anti-diabetes	A combination of resistance exercise and 40 mg/kg/day of saffron administration improved diabetes’ parameters, including insulin release and glucose uptake, in rats.	[20]
Hepatoprotective	20 mg/kg doses of saffron petal hydroalcoholic extracts reduced acetaminophen-induced liver toxicity in rats.	[21]
	100 mg/kg doses of saffron hydro- and alcoholic extracts prevented liver injury in rabbits with prolonged exposure to amiodarone.	[22]
	80 mg/kg ethanolic extracts of saffron significantly reduced hepathic injury biomarkers during exposure to rifampin.	[23]
Anti-carcinogenic	Aqueous saffron extracts achieved a chemopreventive effect in mice. However, this was not consistently dose dependant.	[24]
Neuroprotecive	A 6.5 mg/kg per *os* saffron extract reduced depressive-like behavior in mice during forced swimming. This was suggested to be related to increased monoaminergic neurotransmission activity.	[25]
	The 20, 40, and 80 μg/mL ethanolic saffron extracts increasingly significantly reversed 500-μM corticosterone-induced PC12 cell death. At 1280 μg/mL, extracts progressively increased cytotoxicity.	[26]
Withdrawal management	Daily doses of 60 mg/kg i.p. saffron extract reduced serverity of withdrawal manifestations in adult male rats.	[27]

**Table 2:** Overview of experimental trials on anti-depressant functions of officinal extracts.

Plant	Organisms	Dose	Tests	Parameters	Results	Reference
*Ginkgo biloba*	Male BALB/c mice	17-day dose of 5, 10, 20, 40 mg/kg	Forced swimming	Immobility period, locomotor activity, and monoamines	Mice exposed to 10 mg/kg/day of *G. biloba* extract showed a decline of 39% immobility time after forced swimming test. Reduced lipid peroxidation and radicals were associated with the extract.	[46]
*Ginkgo biloba*	136 Elderly humans with depression	Thrice 19.2 mg/day	Placebo-controlled trial	HAM-D * and serum S100B levels	Those exposed to *G. biloba* scored better on the HAM-D and showed lower expression of serum S100B, a brain injury marker.	[47]
*Hypericum perforatum*	20 Male mice (strain BlC_57_)	Single dose of 7, 35, 70 mg/kg	Forced swimming and tail suspension	Immobility period	Mice displayed a negative correlation between dose (7, 35, and 70 mg/kg b.m.) of St. John’s Wort extract and immobility time after forced swimming and tail suspension.	[48]
*Curcuma longa*	Male Sprague–Dawley rats	14-day dose of 2.5, 5, 10 mg/kg	Forced swimming, olfactory bulbectomy, open field, and passive avoidance test	Various levels of monoamines and metabolites, immobility, and behavioral abnormalities	Curcumin administration reversed neurotransmitter deficits induced by olfactory bulbectomy tests in rats. Behavior after olfactory bulbectomy and forced swimming tests was improved.	[41]
*Valeriana officinalis*	Albino Laca mice	Single- and 14-day admission of 10, 20, 40 mg/kg	Forced swimming	Immobility period, locomotor activity, norepinephrine and dopamine levels	Single administration of dichloromethane extracted from valerian significantly inhibited forced swimming-induced immobility in mice. Additionally, sustained administration decreased immobility and increased norepinephrine and dopamine levels.	[49]
*Hibiscus tiliaceus*	>40 Male Swiss albino mice	Single dose of 3, 10, 30 mg/kg	Forced swimming, tail suspension, and elevated plus-maze	Immobility, maze arm entry	Methanolic hibiscus flower extracts decreased the period of immobility times.	[50]
*Paeonia lactiflora*	80 Male ICR mice	80 and 160 mg/kg, 7 days	Forced swimming and tail suspension	Immobility period and MAO A and B activity	Peony extracts inhibited MAO A and B activity in mouse brains, significantly reduced inmobility times.	[51]
*Piper methysticum*	60 adult Humans	5 doses of 250 mg/day for 3 weeks	Placebo-controlled, double-blind, crossover trial	Hamilton Anxiety Scale, Beck Anxiety Inventory, and Montgomery–Asberg Depression Rating Scale	Aqueous kava extracts reduced all assessed parameters. Additionally, no clinical hepatotoxicity was observed, which has been reason for *P. methysticum*’s withdrawal in some countries.	[52]
*Lavandula angustifolia*	35 Wistar rats	Thrice administered 3428 mg/kg	Forced swimming	Immobility period	Aqueous lavender extracts significantly reduced immobility periods after forced swimming in rats, which was comparable to imipramine (30 mg/kg).	[53]
*Passiflora foetida*	30 Male Swiss albino mice	Single dosage of 100, 200, 300 mg/kg	Forced swimming, tail suspension, and open field	Immobility and locomotor activity	Methanolic passionflower extracts decreased immobility time in a dose-dependent manner in mice after tail suspension and forced swimming. Results were comparable to fluoxetine (20 mg/kg) and imipramine (15 mg/kg). No significant effects were observed on locomotor activity.	[54]

* HAM-D: Hamilton Depression Rating Scale.

**Table 3:** Studies on pharmacological activities relating to anti-depressant-like effects of saffron. BDI, Beck depression inventory; HAE, hydro-alcoholic extract; PCI, percutaneous coronary intervention; CAD, coronary artery disease; HAM-D, Hamilton Depression Rating Scale; PMS: premenstrual syndrome; GAD, generalized anxiety disorder; MMT, methadone maintenance treatment.

Aim of the Research	Type of Study	No. of Patients	Treatment	Time of Treatment (Weeks)	Results	References
Comparison of saffron and imipramine	Double-blind, randomized trial	30	Stigma of saffron, 30 mg/day	6	The effect of stigma of saffron was similar to imipramine in the treatment of mild to moderate depression.	[86]
Hydro-alcoholic extract of saffron versus fluoxetine	Double-blind, randomized pilot trial	40	Stigma of saffron, 30 mg/day	6	The effect of stigma of saffron was similar to fluoxetine in the treatment of mild to moderate depression.	[85]
Saffron (petal) in the treatment of mild to moderate depression	Double-blind, randomized, and placebo-controlled trial	40	Petal of saffron, 30 mg/day	6	The outcome on the HAM-D showed that the petal of saffron could produce a significantly better effect than the placebo.	[109]
Comparison of petal of saffron and fluoxetine	Double-blind, randomized trial	40	Petal of saffron, 15 mg/day (morning and evening)	8	Petal of saffron was found to be similarly effective to fluoxetine in the treatment of mild to moderate depression.	[87,110]
40 and 80 mg HAE of saffron against fluoxetine	Double-blind, randomized, clinical trial	60	Saffron, 40 and 80 mg/day + fluoxetine (30 mg)	6	Effective in treatment of mild to moderate depressive disorders.	[88,111]
Saffron with fluoxetine in PCI patients	Double-blind, randomized, clinical trial	40	Saffron (30mg/day)	6	Effective as fluoxetine (40 mg/day) in improving depressive symptoms of patients who were suffering from major depressive disorder (MDD).	[112]
Saffron and crocin in improving mental and sexual health in CAD patients	Double-blind, placebo-controlled, randomized, clinical trial	58	Stigma of saffron, 30 mg/day OR	8	The outcome of BDI-II scores significantly decreased after 8 weeks of intervention.	[113]
Saffron in the treatment of PMS	Double-blind, randomized, and placebo-controlled trial	50	30 mg, saffron petal during pre-menstrual syndrome	8	The depression measured significantly decreased.	[114]
Saffron versus citalopram in the major depressive disorder with anxious distress	Double-blind, controlled, clinical trial	66	30 mg, saffron stigma	6	Effective against moderate to major depression.	[107]
Saffron as an add-on therapy to sertraline in mild to moderate generalized anxiety disorder	Double-blind, randomized, controlled trial	40	500-mg capsule containing 450 mg of saffron (type not recorded)	6	Decreased mild to moderate generalized anxiety disorder with saffron as well as with sertraline.	[108]
Crocin on depression in subjects with metabolic syndrome	Randomized, double-blind, controlled, clinical trial	33	30 mg, saffron (crocin)	8	Decreased depressive symptoms in patients with metabolic syndrome.	[115]
Saffron improved depression and reduced homocysteine level in patients with major depression	Randomized, double-blind study	40	30 mg, saffron (stigma) and 20 mg, fluoxetine	4	The BDI score decreased in patients with major depression.	[116]
Comparison of saffron versus fluoxetine in treatment of mild to moderate post-partum depression	Double-blind, randomized, clinical trial	60	30 mg, saffron (stigma)	6	Significantly decreased mild to moderate depression and post-menopausal hot flashes.	[110]
Affron^®^, a standardized extract from saffron	Randomised, double-blind, placebo-controlled study	80	14 mg, saffron (stigma)	8	Significant reduction in mild to moderate depression.	[117]
Saffron in the treatment of anxiety and depression	Double-blind, randomized, and placebo- controlled trial	60	100 mg, saffron (stigma)	12	Significant decrease in mild to moderate depression.	[118]
Saffron (petal) in the treatment of mild to moderate depression	Double-blind, randomized, and placebo-controlled trial	36	30 mg, saffron (stigma) and 40 mg, fluoxetine	4	No significant decrease.	[119]
Effects of saffron on depression and lipid profile	Double-blind comparative study	40	30 mg, saffron (petal)	6	Decrease in major depression of those who met DSM-IV criteria.	[109]
Saffron stigma in mothers suffering from mild to moderate post-partum depression	Double-blind, randomized, placebo-controlled trial	40	30 mg, saffron (type not recorded) and 20 mg, fluoxetine	4	Significant decrease in major depression.	[120]
Crocin in major depressive disorder	Randomized, double-blind, placebo-controlled, pilot clinical trial	78	30 mg, saffron (stigma)	8	Significant decrease in mild to moderate depression.	[111]
Crocin on psychological parameters in patients under MMT	Randomized clinical trial	46	30 mg, saffron (crocin) and 20 mg, fluoxetine	4	Significant decrease in major depression.	[121]
Crocin on psychological parameters in patients under MMT	Randomized, double-blind, placebo-controlled trial	50	30 mg per day, saffron (crocin)	8	Improved depression symptoms during methadone maintenance treatment (MMT).	[122]
	Double-blind, randomized, and placebo- controlled trial	28	150 mg per day, saffron	6	Increased serotonin and happiness were further heightened in supplemented group. Anandamide, dopamine, and β-endorphin were significantly increased under suplementeation, whereas placebo remained unchanged.	[123]

**Table 4:** Effect of experimental drug delivery systems on stability, loading, and bioavailability of crocin, as reported in literature.

Matrix	Results	Reference
Chitosan-alginate nanoparticles	Highest crocin loading achieved at pH 1.2 with a biphasic release in simulated gastric fluids. The loaded nanoparticles were equivalent in DPPH free radical scavenging and ferric-reducing ability of plasma as free crocin and exhibited an anti-cancer effect.	[128]
Maltodextrin nanoencapsulates	Nanoencapsulated crocin was more stable at simulated gastrointestinal conditions. While encapsulation increased bioaccessibility (from 61% to 72%), the combination of caffeic acid with encapsulation increased the bioaccessibility to almost 80%.	[129]
Maltodextrin/pectin/whey protein concentrate nanoencapsulates	Combinations of whey protein concentrate and pectin yielded the highest crocin encapsulation efficiencies, exceeding 95%. Thus, minimal amounts of crocins were exposed at the particles’ surfaces. Furthermore, an improved stability against stressors was suggested.	[130]
Chitosan-gum arabic nanoencapsulates	Crocin was encapsulated with an efficiency of 29 to 52%. The release profiles showed an oscillatory relationship with time at pH 1 and 2. This oscillatory relation was suggested to be a result of rapid degradation of released crocin.	[131]
Cholesterol-Tween 40 nanoniosomes	Encapsulation efficiency was 46%, and 61% of crocin was released after 6 h in mice. Intra-arterially injected crocin-laden niosomes decreased ischemic indicator molecules in rats and mitigated I/R tissue damages.	[132]
Bacterial nanocellulose membrane	The nanocellulose membrane exhibited a stable and prolonged transdermal release through mice skin in a Franz diffusion cell.	[133]
Chitosan-alginate	An encapsulation efficiency of 92% was attained. The resulting nanoparticles stabilized crocin degradation at pH 2, enhanced bioavailability, and showed a pH-mediated release.	[134]
Solid lipid nanoparticles	Increased stability, high encapsulation efficiency.	[135]
Selenium nanoparticles	Crocin release rate was pH dependant, with 91% released after 48 h at pH 5.3, whereas just a mere 35% was released at pH 7.4 during the same time. The administration of loaded nanoparticles resulted in enhanced cytotoxicity in lung cancer cells and inhibited tumor growth in a mice model.	[136]
Poly(lactic-co-glycolic acid) nanoparticles	Entrapment efficiency reached 59%, and 78% of crocin was released after 24 h at pH 7.4, sustaining release throughout 48 h. Release was increased at pH 6.5 to 84% after 24 h.	[137]

## Data Availability

Not applicable.

## References

[1] Pandita D., Aftab T., Hakeem K.R. (2021). Saffron (*Crocus sativus* L.): Phytochemistry, therapeutic significance and omics-based biology. Medicinal and Aromatic Plants.

[2] Hosseinzadeh H., Nassiri-Asl M. (2013). Avicenna’s (Ibn Sina) the canon of medicine and saffron (*Crocus sativus*): A review. Phytother. Res..

[3] Christodoulou E., Kadoglou N.P., Kostomitsopoulos N., Valsami G. (2015). Saffron: A natural product with potential pharmaceutical applications. J. Pharm. Pharmacol..

[4] Srivastava T.N., Rajasekharan S., Badola D.P., Shah D.C. (1985). Important medicinal plants of jammu and kashmir I. Kesar (saffron). Anc. Sci. Life.

[5] Basker D., Negbi M. (1983). Uses of saffron. Econ. Bot..

[6] Dhar A.K., Mir G.M. (1997). Saffron in Kashmir-VI: A review of distribution and production. J. Herbs Spices Med. Plants.

[7] Jan S., Wani A.A., Kamili A.N., Kashtwari M. (2014). Distribution, chemical composition and medicinal importance of saffron (*Crocus sativus* L.). Afr. J. Plant Sci..

[8] Ghaffari S.M., Bagheri A. (2010). Stigma variability in saffron (*Crocus sativus* L.). Afr. J. Biotechnol..

[9] Gambella F., Paschino F., Bertetto A.M. (2013). Perspectives in the mechanization of saffron (*Crocus sativus* L.). Int. J. Mech. Control..

[10] Galigani P.F., Pegna F.G., Megbi M. (2006). Mechanized Saffron Cultivation, Including Harvesting. Saffron: Crocus Sativus L..

[11] Bathaie S.Z., Mousavi S.Z. (2011). Historical uses of saffron: Identifying potential new avenues for modern Research. Avicenna J. Phytomed..

[12] Ait-Oubahou A., El-Otmani M., Megbi M. (2006). 8. Saffron Cultivation in Morocco. Saffron: Crocus sativus L..

[13] Shariatifar N., Shoeibi S., Sani M.J., Jamshidi A.H., Zarei A., Mehdizade A., Dadgarnejad M. (2014). Study on diuretic activity of saffron (stigma of *Crocus sativus* L.) Aqueous extract in rat. J. Adv. Pharm. Technol..

[14] Amin B., Hosseinzadeh H., Watson R.R.P., Victor R. (2015). Analgesic and Anti-Inflammatory Effects of *Crocus sativus* L. (Saffron). Bioactive Nutraceuticals and Dietary Supplements in Neurological and Brain Disease: Prevention and Therapy.

[15] Vahidi A.R., Bashardost N., Akhondi H. (2007). The analgesic effect of saffron extract in rats as compared with morphine sulfate. Planta Med..

[16] Hosseinzadeh H., Younesi H.M. (2002). Antinociceptive and anti-inflammatory effects of *Crocus sativus* L. stigma and petal extracts in mice. BMC Pharmacol..

[17] Khan A., Muhamad N.A., Ismail H., Nasir A., Khalil A.A.K., Anwar Y., Khan Z., Ali A., Taha R.M., Al-Shara B. (2020). Potential Nutraceutical Benefits of In Vivo Grown Saffron (*Crocus sativus* L.) As Analgesic, Anti-inflammatory, Anticoagulant, and Antidepressant in Mice. Plants.

[18] Hosseinzadeh H., Khosravan V. (2002). Anticonvulsant effects of aqueous and ethanolic extracts of *Crocus sativus* L. stigmas in mice. Arch. Iran. Med..

[19] Boskabady M.H., Byrami G., Feizpour A. (2014). The effect of safranal, a constituent of *Crocus sativus* (saffron), on tracheal responsiveness, serum levels of cytokines, total NO and nitrite in sensitized guinea pigs. Pharmacol. Rep..

[20] Dehghan F., Hajiaghaalipour F., Yusof A., Muniandy S., Hosseini S.A., Heydari S., Salim L.Z.A., Azarbayjani M.A. (2016). Saffron with resistance exercise improves diabetic parameters through the GLUT4/AMPK pathway in-vitro and in-vivo. Sci. Rep..

[21] Omidi A., Riahinia N., Montazer Torbati M.B., Behdani M.-A. (2014). Hepatoprotective effect of *Crocus sativus* (saffron) petals extract against acetaminophen toxicity in male Wistar rats. Avicenna J. Phytomed..

[22] Saleem N., Ahmad M., Kamran S., Riaz H., Mehmood Y., Raza S. (2016). Hepatoprotective Effect of *Crocus sativus* on Amiodarone-Induced Liver Toxicity. Br. J. Pharm. Res..

[23] Mohajeri D., Rezaei A., Doustar Y., Abbasi M.M. (2011). Hepatoprotective effect of ethanolic extract of saffron stigma in comparison with silymarin against rifampin induced hepatotoxicity in rats. Zahedan. J. Res. Med. Sci..

[24] Premkumar K., Abraham S.K., Santhiya S.T., Ramesh A. (2003). Protective effects of saffron (*Crocus sativus* Linn.) on genotoxins-induced oxidative stress in Swiss albino mice. Phytother. Res..

[25] Monchaux De Oliveira C., Pourtau L., Vancassel S., Pouchieu C., Capuron L., Gaudout D., Castanon N. (2021). Saffron Extract-Induced Improvement of Depressive-Like Behavior in Mice Is Associated with Modulation of Monoaminergic Neurotransmission. Nutrients.

[26] Chen X., Yang T., Zhang C., Ma Z. (2022). RNA-seq based transcriptome analysis of ethanol extract of saffron protective effect against corticosterone-induced PC12 cell injury. BMC Complement. Med. Ther..

[27] Kiashemshaki B., Safakhah H.A., Ghanbari A., Khaleghian A., Miladi-Gorji H. (2021). Saffron (*Crocus sativus* L.) stigma reduces symptoms of morphine-induced dependence and spontaneous withdrawal in rats. Am. J. Drug Alcohol Abuse.

[28] VandenBosch G.R. (2015). APA Dictionary of Psychology.

[29] Fava M., Kendler K.S. (2000). Major Depressive Disorder. Neuron.

[30] Williams J.B.W. (2001). Standardizing the Hamilton Depression Rating Scale: Past, present, and future. Eur. Arch. Psychiatry Clin. Neurosci..

[31] Cuijpers P., Reynolds C.F., Donker T., Li J., Andersson G., Beekman A. (2012). Personalized Treatment of Adult Depression: Medication, Psychotherapy, or Both? A Systematic Review. Depress. Anxiety.

[32] Blier P. (2016). Neurobiology of Depression and Mechanism of Action of Depression Treatments. J. Clin. Psychiatry.

[33] Ostadkarampour M., Putnins E.E. (2021). Monoamine Oxidase Inhibitors: A Review of Their Anti-Inflammatory Therapeutic Potential and Mechanisms of Action. Front. Pharmacol..

[34] Santomauro D.F., Mantilla Herrera A.M., Shadid J., Zheng P., Ashbaugh C., Pigott D.M., Abbafati C., Adolph C., Amlag J.O., Aravkin A.Y. (2021). Global prevalence and burden of depressive and anxiety disorders in 204 countries and territories in 2020 due to the COVID-19 pandemic. Lancet.

[35] Liu L., Liu C., Wang Y., Wang P., Li Y., Li B. (2015). Herbal Medicine for Anxiety, Depression and Insomnia. Curr. Neuropharmacol..

[36] Shahrajabian M.H., Sun W., Cheng Q. (2019). A review of Ginseng species in different regions as a multipurpose herb in traditional Chinese medicine, modern herbology and pharmacological science. J. Med. Plant Res..

[37] Xu C., Teng J., Chen W., Ge Q., Yang Z., Yu C., Yang Z., Jia W. (2010). 20 (S)-protopanaxadiol, an active ginseng metabolite, exhibits strong antidepressant-like effects in animal tests. Prog. Neuro Psychopharmacol. Biol. Psychiatry.

[38] Mao Q.Q., Ip S.P., Xian Y.F., Hu Z., Che C.T. (2012). Anti-depressant-like effect of peony: A mini-review. Pharm. Biol..

[39] Chassagne F., Huang X., Lyles J.T., Quave C.L. (2019). Validation of a 16th century traditional Chinese medicine use of ginkgo biloba as a topical antimicrobial. Front. Microbiol..

[40] Kulkarni S.K., Bhutani M.K., Bishnoi M. (2008). Antidepressant activity of curcumin: Involvement of serotonin and dopamine system. Psychopharmacology.

[41] Xu Y., Ku B.S., Yao H.Y., Lin Y.H., Ma X., Zhang Y.H., Li X.J. (2005). Antidepressant effects of curcumin in the forced swim test and olfactory bulbectomy models of depression in rats. Pharmacol. Biochem. Behav..

[42] Rzhepakovsky I.V., Areshidze D.A., Avanesyan S.S., Grimm W.D., Filatova N.V., Kalinin A.V., Kochergin S.G., Kozlova M.A., Kurchenko V.P., Sizonenko M.N. (2022). Phytochemical Characterization, Antioxidant Activity, and Cytotoxicity of Methanolic Leaf Extract of Chlorophytum Comosum (Green Type) (Thunb.) Jacq. Molecules.

[43] Isemura M., Miyoshi N., Pervin M., Suzuki T., Unno K., Nakamura Y. (2015). Green tea catechins for well-being and therapy: Prospects and opportunities. Botanics.

[44] Pham N.M., Nanri A., Kurotani K., Kuwahara K., Kume A., Sato M., Hayabuchi H., Mizoue T. (2014). Green tea and coffee consumption is inversely associated with depressive symptoms in a Japanese working population. Public Health Nutr..

[45] Bandaruk Y., Mukai R., Kawamura T., Nemoto H., Terao J. (2012). Evaluation of the Inhibitory Effects of Quercetin-Related Flavonoids and Tea Catechins on the Monoamine Oxidase-A Reaction in Mouse Brain Mitochondria. J. Agric. Food Chem..

[46] Rojas P., Serrano-García N., Medina-Campos O.N., Pedraza-Chaverri J., Ögren S.O., Rojas C. (2011). Antidepressant-like effect of a Ginkgo biloba extract (EGb761) in the mouse forced swimming test: Role of oxidative stress. Neurochem. Int..

[47] Dai C.X., Hu C.C., Shang Y.S., Xie J. (2018). Role of Ginkgo biloba extract as an adjunctive treatment of elderly patients with depression and on the expression of serum S100B. Medicine.

[48] Bach-Rojecky L., Kalodera Z., Samaržija I. (2004). The antidepressant activity of Hypericum perforatum L. measured by two experimental methods on mice. Acta Pharm..

[49] Sah S.P., Mathela C.S., Chopra K. (2011). Antidepressant effect of Valeriana wallichii patchouli alcohol chemotype in mice: Behavioural and biochemical evidence. J. Ethnopharmacol..

[50] Vanzella C., Bianchetti P., Sbaraini S., Vanzin S.I., Melecchi M.I.S., Caramão E.B., Siqueira I.R. (2012). Antidepressant-like effects of methanol extract of Hibiscus tiliaceus flowers in mice. BMC Complement. Altern. Med..

[51] Mao Q.Q., Ip S.P., Tsai S.H., Che C.T. (2008). Antidepressant-like effect of peony glycosides in mice. J. Ethnopharmacol..

[52] Sarris J., Kavanagh D.J., Byrne G., Bone K.M., Adams J., Deed G. (2009). The Kava Anxiety Depression Spectrum Study (KADSS): A randomized, placebo-controlled crossover trial using an aqueous extract of Piper methysticum. Psychopharmacology.

[53] Kageyama A., oshio M., Horiuchi H., Yokogoshi H., ueno T., Masuda H., Kageyama A., Yokogosh H. (2012). Antidepressant-like Effects of an Aqueous Extract of Lavender (*Lavandula angustifolia* Mill.) in Rats. Food Sci. Technol. Res..

[54] Santhosh P., Venugopal R., Nilakash S., Kunjbihari S., Mangala L. (2010). Antidepressant activity of methanolic extract of Passiflora foetida leaves in mice. Int. J. Pharm. Pharm..

[55] Kabiri M., Rezadoost H., Ghassempour A. (2017). A comparative quality study of saffron constituents through HPLC and HPTLC methods followed by isolation of crocins and picrocrocin. LTW.

[56] Moratalla-López N., Bagur M.J., Lorenzo C., Martínez-Navarro M.E., Rosario Salinas M., Alonso G.L. (2019). Bioactivity and Bioavailability of the Major Metabolites of *Crocus sativus* L. Flower. Molecules.

[57] Hosseinzadeh H., Sadeghnia H.R. (2005). Safranal, a constituent of *Crocus sativus* (saffron), attenuated cerebral ischemia induced oxidative damage in rat hippocampus. J Pharm. Pharm. Sci..

[58] Hosseinzadeh H., Talebzadeh F. (2005). Anticonvulsant evaluation of safranal and crocin from *Crocus sativus* in mice. Fitoterapia.

[59] Hosseinzadeh H., Sadeghnia H.R. (2007). Protective effect of safranal on pentylenetetrazol-induced seizures in the rat: Involvement of GABAergic and opioids systems. Phytomedicine.

[60] Karimi G.R., Hosseinzadeh H., Hosseinzadeh H., Khaleghpanah P. (2001). Study of antidepressant effect of aqueous and ethanol extract of *Crocus sativus* in mice. Iran. J. Basic Med. Sci..

[61] ChemSpider CSID:55000. http://www.chemspider.com/Chemical-Structure.55000.html.

[62] ChemSpider CSID:4444644. https://www.chemspider.com/Chemical-Structure.4444644.html.

[63] ChemSpider CSID:115678. https://www.chemspider.com/Chemical-Structure.115678.html.

[64] ChemSpider CSID:4444645. https://www.chemspider.com/Chemical-Structure.4444645.html.

[65] Suchareau M., Bordes A., Lemée L. (2021). Improved quantification method of crocins in saffron extract using HPLC-DAD after qualification by HPLC-DAD-MS. Food. Chem..

[66] Zhao C., Kam H.-T., Chen Y., Gong G., Hoi M.P.-M., Skalicka-Woźniak K., Dias A.C.P., Lee S.M.-Y. (2021). Crocetin and Its Glycoside Crocin, Two Bioactive Constituents From *Crocus sativus* L. (Saffron), Differentially Inhibit Angiogenesis by Inhibiting Endothelial Cytoskeleton Organization and Cell Migration Through VEGFR2/SRC/FAK and VEGFR2/MEK/ERK Signaling Pathways. Front. Pharmacol..

[67] Gutheil W.G., Reed G., Ray A., Anant S., Dhar A. (2012). Crocetin: An Agent Derived from Saffron for Prevention and Therapy for Cancer. Curr. Pharm. Biotechnol..

[68] Escribano J., Alonso G.-L., Coca-Prados M., Fernández J.-A. (1996). Crocin, safranal and picrocrocin from saffron (*Crocus sativus* L.) inhibit the growth of human cancer cells in vitro. Cancer Lett..

[69] Xu G.-L., Li G., Ma H.-P., Zhong H., Liu F., Ao G.-Z. (2009). Preventive Effect of Crocin in Inflamed Animals and in LPS-Challenged RAW 264.7 Cells. J. Agric. Food Chem..

[70] Godugu C., Pasari L.P., Khurana A., Anchi P., Saifi M.A., Bansod S.P., Annaldas S. (2020). Crocin, an active constituent of *Crocus sativus* ameliorates cerulein induced pancreatic inflammation and oxidative stress. Phytother. Res..

[71] Georgiadou G., Grivas V., Tarantilis P.A., Pitsikas N. (2014). Crocins, the active constituents of *Crocus sativus* L. counteracted ketamine–induced behavioural deficits in rats. Psychopharmacology.

[72] Hosseinzadeh H., Motamedshariaty V., Hadizadeh F. (2007). Antidepressant effect of keamperol, a constituent of saffron (*Crocus sativus*) petal, in mice and rats. Pharmacologyonline.

[73] Hosseinzadeh H., Karimi G., Niapoor M. (2004). Antidepressant Effect of *Crocus sativus* L. Stigma Extracts and Their Constituents, Crocin and Safranal, in Mice. Acta Hortic..

[74] Xi L., Qian Z. (2006). Pharmacological properties of crocetin and crocin (digentiobiosyl ester of crocetin) from saffron. Nat. Prod. Commun..

[75] Zhang Y., Geng J., Hong Y., Jiao L., Li S., Sun R., Xie Y., Yan C., Aa J., Wang G. (2019). Orally Administered Crocin Protects Against Cerebral Ischemia/Reperfusion Injury Through the Metabolic Transformation of Crocetin by Gut Microbiota. Front. Pharmacol..

[76] Hosseini A., Razavi B.M., Hosseinzadeh H. (2018). Pharmacokinetic Properties of Saffron and its Active Components. Eur. J. Drug. Metab. Pharmacokinet..

[77] Rahaiee S., Moini S., Hashemi M., Shojaosadati S.A. (2015). Evaluation of antioxidant activities of bioactive compounds and various extracts obtained from saffron (*Crocus sativus* L.): A review. J. Food Sci. Technol..

[78] Sharma B., Kumar H., Kaushik P., Mirza R., Awasthi R., Kulkarni G.T., Sarwat M., Sumaiya S. (2020). Therapeutic Benefits of Saffron in Brain Diseases: New Lights on Possible Pharmacological Mechanisms. Saffron: The Age-Old Panacea in a New Light.

[79] Rao S.V., Muralidhara, Yenisetti S.C., Rajini P.S. (2016). Evidence of neuroprotective effects of saffron and crocin in a Drosophila model of parkinsonism. Neurotoxicology.

[80] Moragrega I., Ríos J.L. (2021). Medicinal Plants in the Treatment of Depression: Evidence from Preclinical Studies. Planta Med..

[81] Mokhtari-Zaer A., Saadat S., Ghorani V., Memarzia A., Boskabady M.H., Sarwat M., Sumaiya S. (2020). The Effects of Saffron (*Crocus sativus*) and its Constituents on Immune System. Saffron: The Age-Old Panacea in a New Light.

[82] Fatehi M., Rashidabady T., Hassanabad Z.F. (2007). Effects of Petals Extracts of Saffron on Rat Blood Pressure and on Responses Induced by Electrical Field Stimulation in the Rat Isolated Vas Deferens and Guinea-Pig Ileum. Acta Hortic..

[83] Papandreou M.A., Tsachaki M., Efthimiopoulos S., Cordopatis P., Lamari F.N., Margarity M. (2011). Memory enhancing effects of saffron in aged mice are correlated with antioxidant protection. Behav. Brain Res..

[84] Mousavi S.H., Tayarani N.Z., Parsaee H. (2010). Protective effect of saffron extract and crocin on reactive oxygen species-mediated high glucose-induced toxicity in pc12 cells. Cell. Mol. Neurobiol..

[85] Noorbala A.A., Akhondzadeh S., Tahmacebi-Pour N., Jamshidi A.H. (2005). Hydro-alcoholic extract of *Crocus sativus* L. versus fluoxetine in the treatment of mild to moderate depression: A double-blind, randomized pilot trial. J. Ethnopharmacol..

[86] Akhondzadeh S., Fallah-Pour H., Afkham K., Jamshidi A.H., Khalighi-Cigaroudi F. (2004). Comparison of *Crocus sativus* L. and imipramine in the treatment of mild to moderate depression: A pilot double-blind randomized trial [ISRCTN45683816]. BMC Complement. Altern. Med..

[87] Akhondzadeh Basti A., Moshiri E., Noorbala A.A., Jamshidi A.H., Abbasi S.H., Akhondzadeh S. (2007). Comparison of petal of *Crocus sativus* L. and fluoxetine in the treatment of depressed outpatients: A pilot double-blind randomized trial. Prog. Neuro Psychopharmacol. Biol. Psychiatry.

[88] Moosavi S.M., Ahmadi M., Amini M., Vazirzadeh B. (2014). The effects of 40 and 80 mg hydro- alcoholic extract of *Crocus sativus* in the treatment of mild to moderate depression. J. Maz. Univ. Med. Sci..

[89] Pietikainen P., Pietikainen P. (2018). The Medical Management of Madness. Madness: A History.

[90] De Monte C., Carradori S., Chimenti P., Secci D., Mannina L., Alcaro F., Petzer A., N’Da C.I., Gidaro M.C., Costa G. (2014). New insights into the biological properties of *Crocus sativus* L.: Chemical modifications, human monoamine oxidases inhibition and molecular modeling studies. Eur. J. Med. Chem..

[91] Dai L., Chen L., Wang W. (2020). Safety and Efficacy of Saffron (*Crocus sativus* L.) for Treating Mild to Moderate Depression: A Systematic Review and Meta-analysis. J. Nerv. Ment. Dis..

[92] Srivastava R., Ahmed H., Dixit R., Dharamveer, Saraf S. (2010). Crocus sativus L.: A comprehensive review. Pharmacogn. Rev..

[93] Shafiee M., Arekhi S., Omranzadeh A., Sahebkar A. (2018). Saffron in the treatment of depression, anxiety and other mental disorders: Current evidence and potential mechanisms of action. J. Affect. Disord..

[94] Kawabata K., Tung N.H., Shoyama Y., Sugie S., Mori T., Tanaka T. (2012). Dietary crocin inhibits colitis and colitis-associated colorectal carcinogenesis in male ICR mice. Evid. Based Complement. Altern..

[95] Dwivedi Y. (2009). Brain-derived neurotrophic factor: Role in depression and suicide. Neuropsychiatr. Dis. Treat..

[96] Wang J.Q., Mao L. (2019). The ERK Pathway: Molecular Mechanisms and Treatment of Depression. Mol. Neurobiol..

[97] Wang J.Q., Fibuch E.E., Mao L. (2007). Regulation of mitogen-activated protein kinases by glutamate receptors. J. Neurochem..

[98] Lu L., Wu D., Wang K., Tang J., Chen G. (2020). Beneficial Effects of Crocin against Depression via Pituitary Adenylate Cyclase-Activating Polypeptide. Biomed. Res. Int..

[99] Hausenblas H.A., Saha D., Dubyak P.J., Anton S.D. (2013). Saffron (*Crocus sativus* L.) and major depressive disorder: A meta-analysis of randomized clinical trials. J. Integr. Med..

[100] Ettehadi H., Mojabi S.N., Ranjbaran M., Shams J., Sahraei H., Hedayati M., Asefi F. (2013). Aqueous Extract of Saffron (*Crocus sativus*) Increases Brain Dopamine and Glutamate Concentrations in Rats. Behav. Brain Sci..

[101] Mohajeri S.A., Sepahi S., Azam A.G., Koocheki A., Khajeh-Hosseini M. (2020). Chapter 27—Antidepressant and antianxiety properties of saffron. Saffron Science, Technology and Health.

[102] Lechtenberg M., Schepmann D., Niehues M., Hellenbrand N., Wünsch B., Hensel A. (2008). Quality and functionality of saffron: Quality control, species assortment and affinity of extract and isolated saffron compounds to NMDA and σ1 (Sigma-1) receptors. Planta Med..

[103] Izquierdo I., da Cunha C., Rosat R., Jerusalinsky D., Ferreira M.B.C., Medina J.H. (1992). Neurotransmitter receptors involved in post-training memory processing by the amygdala, medial septum, and hippocampus of the rat. Neurosci. Biobehav. Rev..

[104] Pitsikas N., Sakellaridis N. (2006). *Crocus sativus* L. extracts antagonize memory impairments in different behavioural tasks in the rat. Behav. Brain Res..

[105] Ghadami M.R., Pourmotabbed A. (2009). The effect of crocin on scopolamine induced spatial learning and memory deficits in rats. Physiol. Pharmacol..

[106] Khaksarian M., Behzadifar M., Behzadifar M., Alipour M., Jahanpanah F., Re T.S., Firenzuoli F., Zerbetto R., Bragazzi N.L. (2019). The efficacy of *Crocus sativus* (Saffron) versus placebo and Fluoxetine in treating depression: A systematic review and meta-analysis. Psychol. Res. Behav. Manag..

[107] Ghajar A., Neishabouri S.M., Velayati N., Jahangard L., Matinnia N., Haghighi M., Ghaleiha A., Afarideh M., Salimi S., Meysamie A. (2017). *Crocus sativus* L versus Citalopram in the Treatment of Major Depressive Disorder with Anxious Distress: A Double-Blind, Controlled Clinical Trial. Pharmacopsychiatry.

[108] Jafarnia N., Ghorbani Z., Nokhostin M., Manayi A., Nourimajd S., Razeghi Jahromi S. (2017). Effect of Saffron (Crocus Satious L.) as an Add-On Therapy to Sertraline in Mild to Moderate Generalized Anxiety Disorder: A Double Blind Randomized Controlled Trial. Arch. Neurosci..

[109] Moshiri E., Basti A.A., Noorbala A.A., Jamshidi A.H., Hesameddin Abbasi S., Akhondzadeh S. (2006). *Crocus sativus* L. (petal) in the treatment of mild-to-moderate depression: A double-blind, randomized and placebo-controlled trial. Phytomedicine.

[110] Kashani L., Esalatmanesh S., Eftekhari F., Salimi S., Foroughifar T., Etesam F., Safiaghdam H., Moazen-Zadeh E., Akhondzadeh S. (2018). Efficacy of *Crocus sativus* (saffron) in treatment of major depressive disorder associated with post-menopausal hot flashes: A double-blind, randomized, placebo-controlled trial. Arch. Gynecol. Obstet..

[111] Tabeshpour J., Sobhani F., Sadjadi S.A., Hosseinzadeh H., Mohajeri S.A., Rajabi O., Taherzadeh Z., Eslami S. (2017). A double-blind, randomized, placebo-controlled trial of saffron stigma (*Crocus sativus* L.) in mothers suffering from mild-to-moderate postpartum depression. Phytomedicine.

[112] Shahmansouri N., Farokhnia M., Abbasi S.H., Kassaian S.E., Noorbala Tafti A.A., Gougol A., Yekehtaz H., Forghani S., Mahmoodian M., Saroukhani S. (2014). A randomized, double-blind, clinical trial comparing the efficacy and safety of *Crocus sativus* L. with fluoxetine for improving mild to moderate depression in post percutaneous coronary intervention patients. J. Affect. Disord..

[113] Abedimanesh N., Ostadrahimi A., Bathaie S.Z., Abedimanesh S., Motlagh B., Jafarabadi M.A., Sadeghi M.T. (2017). Effects of saffron aqueous extract and its main constituent, crocin, on health-related quality of life, depression, and sexual desire in coronary artery disease patients: A double-blind, placebo-controlled, randomized clinical trial. Iran. Red Crescent Med. J..

[114] Agha-Hosseini M., Kashani L., Aleyaseen A., Ghoreishi A., Rahmanpour H., Zarrinara A.R., Akhondzadeh S. (2008). *Crocus sativus* L. (saffron) in the treatment of premenstrual syndrome: A double-blind, randomised and placebo-controlled trial. BJOG Int. J. Obstet..

[115] Jam I.N., Sahebkar A.H., Eslami S., Mokhber N., Nosrati M., Khademi M., Foroutan-Tanha M., Ghayour-Mobarhan M., Hadizadeh F., Ferns G. (2017). The effects of crocin on the symptoms of depression in subjects with metabolic syndrome. Adv. Clin. Exp. Med..

[116] Jelodar G., Javid Z., Sahraian A., Jelodar S. (2018). Saffron improved depression and reduced homocysteine level in patients with major depression: A Randomized, double-blind study. Avicenna J. Phytomed..

[117] Lopresti A.L., Drummond P.D., Inarejos-García A.M., Prodanov M. (2018). Affron^®^, a standardised extract from saffron (*Crocus sativus* L.) for the treatment of youth anxiety and depressive symptoms: A randomised, double-blind, placebo-controlled study. J. Affect. Disord..

[118] Mazidi M., Shemshian M., Mousavi S.H., Norouzy A., Kermani T., Moghiman T., Sadeghi A., Mokhber N., Ghayour-Mobarhan M., Ferns G.A.A. (2016). A double-blind, randomized and placebo-controlled trial of Saffron (*Crocus sativus* L.) in the treatment of anxiety and depression. J. Complement. Integr..

[119] Modabbernia A., Sohrabi H., Nasehi A.A., Raisi F., Saroukhani S., Jamshidi A.H., Tabrizi M., Ashrafi M., Akhondzadeh S. (2012). Effect of saffron on fluoxetine-induced sexual impairment in men: Randomized double-blind placebo-controlled trial. Psychopharmacology.

[120] Sahraian A., Jelodar S., Javid Z., Mowla A., Ahmadzadeh L. (2016). Study the effects of saffron on depression and lipid profiles: A double blind comparative study. Asian. J. Psychiatr..

[121] Talaei A., Hassanpour Moghadam M., Sajadi Tabassi S.A., Mohajeri S.A. (2014). Crocin, the main active saffron constituent, as an adjunctive treatment in major depressive disorder: A randomized, double-blind, placebo-controlled, pilot clinical trial. J. Affect. Disord..

[122] Khalatbari-Mohseni A., Banafshe H.R., Mirhosseini N., Asemi Z., Ghaderi A., Omidi A. (2019). The effects of crocin on psychological parameters in patients under methadone maintenance treatment: A randomized clinical trial. Subst. Abuse Treat. Prev. Policy.

[123] Moghadam B.H., Bagheri R., Roozbeh B., Ashtary-Larky D., Gaeini A.A., Dutheil F., Wong A. (2021). Impact of saffron (*Crocus sativus* Linn) supplementation and resistance training on markers implicated in depression and happiness levels in untrained young males. Physiol. Behav..

[124] Asai A., Nakano T., Takahashi M., Nagao A. (2005). Orally Administered Crocetin and Crocins Are Absorbed into Blood Plasma as Crocetin and Its Glucuronide Conjugates in Mice. J. Agric. Food Chem..

[125] Xi L., Qian Z., Du P., Fu J. (2007). Pharmacokinetic properties of crocin (crocetin digentiobiose ester) following oral administration in rats. Phytomedicine.

[126] Karkoula E., Lemonakis N., Kokras N., Dalla C., Gikas E., Skaltsounis A.-L., Tsarbopoulos A. (2018). Trans-crocin 4 is not hydrolyzed to crocetin following i.p. administration in mice, while it shows penetration through the blood brain barrier. Fitoterapia.

[127] Siddiqui S.A., Blinov A.V., Serov A.V., Gvozdenko A.A., Kravtsov A.A., Nagdalian A.A., Raffa V.V., Maglakelidze D.G., Blinova A.A., Kobina A.V. (2021). Effect of Selenium Nanoparticles on Germination of Hordéum Vulgáre Barley Seeds. Coatings.

[128] Rahaiee S., Hashemi M., Shojaosadati S.A., Moini S., Razavi S.H. (2017). Nanoparticles based on crocin loaded chitosan-alginate biopolymers: Antioxidant activities, bioavailability and anticancer properties. Int. J. Biol. Macromol..

[129] Kyriakoudi A., Tsimidou M.Z. (2018). Properties of encapsulated saffron extracts in maltodextrin using the Büchi B-90 nano spray-dryer. Food Chem..

[130] Esfanjani A.F., Jafari S.M., Assadpoor E., Mohammadi A. (2015). Nano-encapsulation of saffron extract through double-layered multiple emulsions of pectin and whey protein concentrate. J. Food Eng..

[131] Rajabi H., Jafari S.M., Rajabzadeh G., Sarfarazi M., Sedaghati S. (2019). Chitosan-gum Arabic complex nanocarriers for encapsulation of saffron bioactive components. Colloids Surf. A: Physicochem. Eng. Asp..

[132] Naderi R., Pardakhty A., Abbasi M.F., Ranjbar M., Iranpour M. (2021). Preparation and evaluation of crocin loaded in nanoniosomes and their effects on ischemia–reperfusion injuries in rat kidney. Sci. Rep..

[133] Abba M., Ibrahim Z., Chong C.S., Zawawi N.A., Kadir M.R.A., Yusof A.H.M., Razak S.I.A. (2019). Transdermal Delivery of Crocin Using Bacterial Nanocellulose Membrane. Fibers Polym..

[134] Nasrpour S., Yousefi G., Niakosari M., Aminlari M. (2022). Nanoencapsulation of saffron crocin into chitosan/alginate interpolyelectrolyte complexes for oral delivery: A Taguchi approach to design optimization. J. Food Sci..

[135] Puglia C., Santonocito D., Musumeci T., Cardile V., Graziano A.C.E., Salerno L., Raciti G., Crasci L., Panico A.M., Puglisi G. (2019). Nanotechnological Approach to Increase the Antioxidant and Cytotoxic Efficacy of Crocin and Crocetin. Planta Med..

[136] Mary T.A., Shanthi K., Vimala K., Soundarapandian K. (2016). PEG functionalized selenium nanoparticles as a carrier of crocin to achieve anticancer synergism. RSC Adv..

[137] Khan I., Joshi G., Sarkar B., Nakhate K.T., Ajazuddin, Mantha A.K., Kumar R., Kaul A., Chaturvedi S., Mishra A.K. (2020). Doxorubicin and Crocin Co-delivery by Polymeric Nanoparticles for Enhanced Anticancer Potential In Vitro and In Vivo. ACS Appl. Bio Mater..

